# Experiences of a Community-Based Lymphedema Management Program for Lymphatic Filariasis in Odisha State, India: An Analysis of Focus Group Discussions with Patients, Families, Community Members and Program Volunteers

**DOI:** 10.1371/journal.pntd.0004424

**Published:** 2016-02-05

**Authors:** Tali Cassidy, Caitlin M. Worrell, Kristen Little, Aishya Prakash, Inakhi Patra, Jonathan Rout, LeAnne M. Fox

**Affiliations:** 1 School of Public Health and Family Medicine, University of Cape Town, Cape Town, South Africa; 2 Parasitic Diseases Branch, Division of Parasitic Diseases and Malaria, Center for Global Health, Centers for Disease Control and Prevention, Atlanta, Georgia, United States of America; 3 Church's Auxiliary for Social Action, Pusa Road, New Delhi, India; Michigan State University, UNITED STATES

## Abstract

**Background:**

Globally 68 million people are infected with lymphatic filariasis (LF), 17 million of whom have lymphedema. This study explores the effects of a lymphedema management program in Odisha State, India on morbidity and psychosocial effects associated with lymphedema.

**Methodology/Principal Findings:**

Focus groups were held with patients (eight groups, separated by gender), their family members (eight groups), community members (four groups) and program volunteers (four groups) who had participated in a lymphedema management program for the past three years. Significant social, physical, and economic difficulties were described by patients and family members, including marriageability, social stigma, and lost workdays. However, the positive impact of the lymphedema management program was also emphasized, and many family and community members indicated that community members were accepting of patients and had some improved understanding of the etiology of the disease. Program volunteers and community members stressed the role that the program had played in educating people, though interestingly, local explanations and treatments appear to coexist with knowledge of biomedical treatments and the mosquito vector.

**Conclusions/Significance:**

Local and biomedical understandings of disease can co-exist and do not preclude individuals from participating in biomedical interventions, specifically lymphedema management for those with lymphatic filariasis. There is a continued need for gender-specific psychosocial support groups to address issues particular to men and women as well as a continued need for improved economic opportunities for LF-affected patients. There is an urgent need to scale up LF-related morbidity management programs to reduce the suffering of people affected by LF.

## Author Summary

Around the world 68 million people are infected with lymphatic filariasis (LF), a mosquito-borne disease caused by filarial worms. The parasite can damage patients’ lymphatic systems causing pain, infections and swollen limbs, known as lymphedema or, in more advanced stages, elephantiasis. Lymphedema management programs can help patients to deal with the physical and emotional effects of lymphedema and elephantiasis. We held a total of 24 focus groups to discuss the experiences of people with lymphedema in Odisha State, India who participate in such a program. Discussions were held with patients, family members of patients, community members and program volunteers. Significant social, physical and economic difficulties were described by patients and family members, including marriage-related issues, social stigma, and lost workdays. However, the positive impacts of the lymphedema management program were also emphasized, and many family and community members indicated that community members were accepting of patients and had some improved understanding of the cause of the disease. People were able to hold both a ‘scientific’ and a ‘traditional’ understanding of LF at the same time. The financial hardships that people described highlight the need for improved economic opportunities for lymphedema-affected patients. Support programs based in the community also have clear social and emotional benefits. There is an urgent need to scale up LF management programs to reduce the suffering of people affected by LF.

## Introduction

Lymphatic filariasis (LF) is a mosquito-borne disease caused by filarial worms with approximately 68 million people infected and an estimated 17 million suffering from lymphedema globally [1]. India constitutes 42% of the global burden of LF. [1] Disease associated with LF infection can be either acute or chronic. Chronic disease in men commonly manifests as scrotal swelling, known as hydrocele, while both men and women can develop lymphedema or elephantiasis of the limbs. Adenolymphangitis (ADL) episodes, also called acute attacks, are characterized by fever, swelling, and inflammation of the limb and can lead to the progression of lymphedema in affected individuals. [2]

It is well documented that chronic and acute manifestations of LF cause physical suffering, psychosocial effects on individuals, and economic burden to families. [3–6] The same is true of lymphedema caused by podoconiosis, which also typically infects low-income, rural populations. [7–11] Documented psychosocial impacts include feelings of isolation and exclusion from community events, [12] issues around gender, marriage and shame, [8,13,14] and stigma stemming from misconceptions about lymphedema. [15,16]

Anti-filarial medication can help interrupt transmission of LF by clearing microfilaria from the peripheral blood of infected hosts, but has limited effect on reducing or preventing lymphedema in the host. [17] Morbidity management and disability prevention (MMDP) programs are therefore required to address the needs of those experiencing symptoms of LF-related disease. [18] The WHO strategy to address MMDP includes hygiene, skincare, lymphedema-reducing exercise, and elevation activities, and in the case of hydrocele, surgery. [19]

The impact of MMDP programs on the health and functioning of lymphedema patients has been documented using a variety of surveys assessing quality of life, [20,21] but these generic surveys can lack sensitivity to the particular effects of lymphedema in every context. [22] In particular, they do not always measure the social transformation that an MMDP program can bring to modulate the psychosocial effects of lymphedema [23], and the motivation and empowerment of patients to manage their own illness [24]. This study thus adds to a previous evaluation that utilized the WHODAS tool to assess the impact on perceived disability of a community-based lymphedema management program in Odisha state, India. [25] We performed a qualitative evaluation of lymphedema patients involved in a community-based lymphedema management program to assess perceptions of lymphedema and retrospectively evaluate the impact of this program on patients, family members, program volunteers, and community members.

## Methods

### Ethics Statement

This project was submitted for human subjects review to the Center for Global Health at the Centers for Disease Control and Prevention (CDC), Atlanta, Georgia, USA. The project was determined to be program evaluation under CDC policy prior to the implementation of the survey. Permission for the evaluation was obtained from the Odisha State Department of Health and Family Welfare, who also approved the consent process. Participants were asked to give their written informed consent prior to participation. For those unable to write, consent was documented by recording the person's fingerprint or marking the signature line with an ‘X’ and by countersignature of survey personnel.

### Study Area

This program evaluation was conducted in Bolagarh sub-district of Khurda district in Odisha State, India, a region highly endemic for lymphatic filariasis caused by *W*. *bancrofti*. [26] In 2009–2011, the Church’s Auxiliary for Social Action (CASA), an Indian non-governmental organization, implemented active case finding for lymphedema patients and a lymphedema management program in this district which focused on teaching hygiene and limb care for > 20,000 lymphedema patients in Khurda district. In addition, they performed community outreach activities including street plays, radio spots, and distributed informational brochures to teach people about LF and lymphedema care. A longitudinal quantitative evaluation of perceived disability by patients in the program was performed from July 2009-July 2011, the details of which are discussed in a separate manuscript. [25]

### Study Design

Focus groups were held with lymphedema patients, their families, program volunteers, and community members. Participants were drawn from 11 different villages and represented a convenience sample, selected and invited with the help of CASA to incorporate a range of perspectives and areas.

Discussion guides and probes were created in the local language, Oriya, and reviewed with the interviewers as part of their training. Interviewers and note takers also practiced facilitation and note-taking as a group so that the trainers could provide feedback, focusing on prompts and encouraging participation among all group members. The discussion guides were subsequently piloted with both men and women with lymphedema in Odisha State to assure understanding and test the suitability of each question.

Focus group discussions were conducted in Oriya and took place in an easily accessible, central location in the community, such as a house of worship, classroom, volunteers’ home, or local business. Each focus group took approximately one hour. Basic information about each participant was gathered including age, sex, occupation, and participation in mass drug administration (MDA). The patient, family member, and community member focus groups were sex segregated to encourage open discussion. The focus group discussion team consisted of a moderator, who guided group discussion between six and 12 individuals in Oriya, and a note taker. Discussions were electronically recorded and the moderator and note taker subsequently expanded and transcribed the remarks. Together the moderator and note taker, who were fluent in both Oriya and English, also translated the transcript into English.

### Patients

Eight sex-segregated focus group discussions were held with patients. Program participants were assessed for lymphedema stage according to the Dreyer scale by an experienced grader. [27] They were also asked to recall the number of years affected by lymphedema and length of participation in the program. The discussion was designed to elicit information on the following issues: (i) lymphedema causes and treatment; (ii) experiences and challenges of living with lymphedema including social, economic, and psychological aspects, and (iii) experiences and effects of participating in the lymphedema management program.

### Patient Family Members

Eight sex-segregated focus group discussions were held with family members of individuals with lymphedema. The discussion was designed to elicit information on the following issues: (i) experiences living with a family member with lymphedema, including how such families are treated; (ii) experiences and challenges of caring for their family member with lymphedema; and (iii) effect of participating in the lymphedema management program on the family.

### Program Volunteers

Four mixed-sex focus group discussions were held with CASA program volunteers. The discussion was designed to elicit information on the following issues: (i) motivations to volunteer for the program; (ii) experiences with the lymphedema management program, and (iii) challenges faced both by volunteers as well as individuals receiving treatment.

### Community Members

Four focus group discussions (three sex-segregated, one mixed) were held with community members in villages where the lymphedema management program was conducted. The discussion was designed to elicit information on the following issues: (i) lymphedema causes and treatment; (ii) experiences and challenges faced by individuals affected by lymphedema, and (iii) interactions and experiences with the lymphedema management program.

### Data Management and Analysis

Participants were assigned a unique identification code, linking their demographic information with their responses. Anonymized focus group transcriptions were kept in separate Microsoft Word documents and explored for themes. After an initial evaluation, relevant sections and quotes were transferred into a mind-mapping application [28] and grouped under identified themes. Recurring topics were explored more in depth by revisiting the original transcripts and grouping relevant quotes in Microsoft Excel.

## Results

### Demographic and Clinical Characteristics

Overall, 38 female patients and 36 male patients participated. A total of 74 family members of lymphedema patients as well as 28 program volunteers and 35 community members were involved in focus group discussions.

Demographic characteristics of focus group participants are presented in Table 1. Among male patients, family members and community members, the majority reported their occupation as farmers, while among females in all groups the most common reported profession was housework. Most patients (92%), family members (73%) and program volunteers (96%) had participated in the most recent MDA, whereas only 31% of community members reported participation.

**Table 1:** Demographic characteristics of focus group participants (N = 211).

Characteristic	Patients (N = 74)	Family Members (N = 74)	Program Volunteers (N = 28)	Community Members (N = 35)
Age (mean, range)	62.4 (37–85)	46.6 (18–77)	29.1 (19–47)	41.4 (18–63)
Male gender	36 (49%)	37 (50%)	17 (61%)	22 (63%)
Occupation				
Male				
Business	-	3 (8.1%)	5 (29.4%)	3 (13.6%)
Farmer	25 (69.4%)	17 (45.9%)	4 (23.5%)	8 (36.4%)
Laborer	3 (8.3%)	2 (5.4%)	-	1 (4.5%)
Student	-	1 (2.7%)	3 (17.6%)	-
Weaver	-	6 (16.2%)	-	3 (13.6%)
Other	3 (8.3%)	7 (18.9%)	2 (11.8%)	6 (27.3%)
Unemployed	-	1 (2.7%)	2 (11.8%)	1 (4.5%)
Missing	5 (13.9%)	-	1 (5.9%)	-
Female				
Housework	36 (94.7%)	32 (86.5%)	6 (54.5%)	11 (84.6%)
Student	-	-	3 (27.3%)	-
Other	1 (2.6%)	1 (2.7%)	2 (18.2%)	1 (7.7%)
Missing	1 (2.6%)	4 (10.8%)	-	1 (7.7%)
Participated in Last MDA	68 (92%)	54 (73%)	27 (96%)	11 (31%)

Program volunteers who participated in the study were responsible for an average of 20 patients and made an average of three visits to patients each week.

The clinical characteristics of patients participating in the focus groups are presented in Table 2. Patients had experienced lymphedema for an average of 29.1 years and the average length of participation in the program was 3.3 years. Sixty-nine percent of patients had unilateral lymphedema while 31% had bilateral lymphedema. The most common stage of lymphedema was stage two (54.1%), followed by stage three (28.4%) and clinical characteristics were similar for men and women.

**Table 2:** Clinical characteristics of focus group patients (N = 74).

Clinical Characteristics	Patients (N = 74)	Females (N = 38)	Males (N = 36)
Years with lymphedema (mean, range)	29 (5–73)	26 (5–60)	26 (10–73)
Years in lymphedema management program (mean, range)	3.3 (1.3–5.5)	3.4 (1.2–5.5)	3.3 (2–5)
Location of lymphedema			
Unilateral	51 (69%)	26 (68%)	25 (69%)
Bilateral	23 (31%)	12 (32%)	11 (31%)
Stage of lymphedema^†^			
2	40 (54.1%)	20 (53%)	20 (56%)
3	21 (28.4%)	11 (29%)	10 (28%)
4	3 (4.1%)	1 (3%)	2 (6%)
5	1 (1.4%)	1 (3%)	0 (0%)
6	9 (12.2%)	5 (13%)	4 (11%)

^†^In patients with bilateral lymphedema, stage of lymphedema was determined by the leg with the most advanced stage of lymphedema

### Focus Group Results

Responses to focus group questions are grouped below under themes emerging from the mindmapping process.

#### Assessment of knowledge and awareness of lymphatic filariasis and lymphedema

Both patients and community members were asked about the causes and prevention of LF and lymphedema. In Oriya, there was one word ‘batajoro’ which described both LF infection and the disease, lymphedema or elephantiasis. Although participants had difficulty demonstrating a clear understanding of the underlying biological mechanisms of filarial infection or development of lymphedema, basic preventative measures such as MDA with diethylcarbamazine (DEC), mosquito nets, and lymphedema management techniques were discussed in all focus groups.

Among patients, for example, a 60 year old male farmer (bilateral, stage 3) expounded:

‘Take MDA tablets. It is very helpful. We also have to make people aware not to throw away the pills. Every age group should take the medicine then we prevent LF’.

When asked about management of ‘batajoro’, the majority of patients listed washing and elevating affected limbs and taking pain medication for acute episodes of ADL. A 75 year old female housewife (unilateral, stage 3) said

‘We learned from meeting to elevate the legs in the bed while sleeping.’

When asked about prevention of ‘batajoro’ the majority of community members’ responses mentioned either mosquito nets or medication. A 45 year old housewife said lymphedema could be avoided

‘if we take medicines regularly, use mosquito net’.

Other community responses all mentioned cleanliness, usually in relation to mosquito control, such as a 51 year old housewife stating:

‘If we properly clean the surrounding where we live, that can stop the breeding of mosquito’.

Despite these responses, many patients and community members expounded other causes for ‘batajaro’. Nevertheless, alternative explanations were generally voiced alongside responses that emphasized program messages. For example, claims about dietary, hereditary, or lifestyle causes and treatment of LF and lymphedema were common. This point is illustrated by the comments of one 74 year old farmer (unilateral, stage 2) who claimed rice water and dry fish caused the disease, but later went on to state that he had gotten LF because he had not slept under a mosquito net.

Participants in two of the four community member focus groups mentioned that lymphedema was hereditary, but no disagreement was voiced when others mentioned mosquito transmission causing LF. For example, a 34 year old businessman and community member who stated lymphedema was hereditary later stated that one could prevent him/herself from getting lymphedema by taking medicines regularly.

With regard to treatment, other remedies, including homeopathic medication were mentioned, though most patients claimed it had not worked. Nevertheless, some patients felt that homeopathic treatments did work, such as one 65 year old male farmer (unilateral, stage 2) who asserted:

‘I tried a lot to prevent myself from this disease but nothing happened. One person suggested me to take Ghee [clarified butter] and black pepper. Since then for the past 20 years I am taking and I am not having fever. When I stop taking I get fever. So I think a healthy person should take ghee and black pepper to prevent them from getting Lymphedema.’

#### Impact of lymphedema and acute episodes on patients and family

Patients and family members were asked how the disease impacted their lives. The main themes that surfaced involved the inability to work, especially during periodic acute episodes and the economic impact of this. A 45 year old housewife (patient, bilateral, stage 2) explained that she was not able to do any work in the agriculture field or outside. and another patient (aged 48, unilateral, stage 2) elaborated

‘I cannot walk very long distance, with the pain I am bound to do my house hold work for the family’.

Working hard was identified as a specific risk factor for acute episodes, with statements like

‘If we work hard then we get fever so we are afraid to work’ (from a 45 year old male farmer, unilateral, stage 2).

Not only were economic effects noted at the individual level, but participants claimed that the economic impact extended to the entire family. A 70 year old housewife (unilateral stage 2) illustrated the toll of her lymphedema on other family members:

‘Fever comes when we work hard, and we feel very weak during fever, I take help from my children.’

In a family focus group, a 48 year old male farmer echoed this:

‘during the [acute] attack the whole family suffers. We all have to look after the patient. Two to three persons have to be always with the patient leaving all their work behind’.

Another male family member (35 year old weaver) stated:

‘while my mother suffers from fever. It is very painful for our entire family

…I am not able to concentrate in my work…. This is affecting my work a lot. Even economically I have become very poor’.

Men’s illness was perceived to have greater financial consequences for the family:

‘The male persons with LF have to go out for work to earn for the family, so when they cannot work it affects the entire family’ (60 year old male).

In addition, the emotional impact of the disease was discussed. Patients expressed feelings of social isolation living with lymphedema. As a 48 year old housewife (bilateral, stage 4) put it,

‘We feel bad when people call us as “GODARI” [Elephantiasis], and discuss our swelling legs mischievously.’

Although many patients said their families were supportive, an 80 year old farmer (unilateral, stage 2) divulged:

‘My family members don’t take meals with me. I have to eat separately. This is really hurting me’

A few patients indicated resignation to continual suffering. One 40 year old housewife with stage six lymphedema stated that this was their destiny and a 62 year old male with bilateral stage six lymphedema expressed:

‘I am not able to do anything. My family members are asking when I will die’.

Family members expressed distress at the physical pain and suffering that the patients experience. For example, one unemployed 35 year old man stated of his mother

‘We as family members are mentally depressed when she gets an acute attack’.

A 41 year old housewife explained:

‘Many times we have gone through mental and physical pressure [because of her family member’s lymphedema]’.

#### Experiences with the lymphedema management program

When asked about their experiences with the community-based lymphedema management program, patients credited the program with changing their lives through knowledge and skills development, decreased acute episodes, and increased work productivity. The patients who commented on this question all either enumerated things they had learned or explained how the program had improved their lives. A 37 year old housewife (unilateral, stage 3) addressed both points:

‘I learned about the process of washing legs, applying ointment regularly, use of shoes, elevating the legs while sleeping. I feel better doing all these.’

A patient who had been involved in the program for five years said

‘I have taken the tablets [DEC] for four years and for four years I am free from fever’. (78 year old male, unilateral stage 3)

A 70 year old male farmer with bilateral stage three lymphedema had been involved in the program for three years and claimed not to have had an acute episode throughout the period. Another male farmer (aged 76, unilateral, stage 3) stated

‘my health condition is good due to this program’

and a third 71 year old farmer (unilateral, stage 2) said simply that he was now able to work. Patients appreciated learning the home-based lymphedema management techniques and receiving the patient kit, consisting of a towel, soap, and antifungal ointment, as evidenced by statements such as

‘I am really thankful for the [program] team members; before that we were not aware about LF and the treatment, but now all of us are doing the home-based lymphedema management and feel the change’. (67 year old male farmer, unilateral, stage 3)

Family members repeated similar sentiments. For example, a male farmer stated

‘this program is very helpful for us as well as for the patient. We have learned many things to take care of the patient’.

For the program volunteers, the benefits of the program were felt in the rapport and good relationships they developed with families and patients as well as in what they learned from the program. As a 31 year old male task force member said

‘In this program I learned so many things and we have good relationships with patients and patients’ family members.’

They discussed the socially rewarding nature of their work and how they are needed by the patients and family members, especially during acute episodes and to teach limb washing to the patients and family members.

One 32 year old male volunteer explained that during acute attacks their support was needed, and another 45 year old male recounted:

‘One of the patients’ families of my village disagreed with taking DEC tablets, and then I suggested taking the tablet. So they asked the drug distributor and finally accepted it. Now the patient suffers less from acute attacks’.

For the program volunteers, memorable moments involved displays of gratitude from patients and families and the close relationships they developed with the patients. For example, one 22 year old program volunteer stated

‘one old patient emotionally behaves towards me as more than [his] own daughter.’

Volunteers’ motivations for joining the program varied, however personal and familial pride as well as a religious call to service were commonly described. Several participants indicated family pride connected to community appreciation, with phrases such as

‘My family members are very supportive; they feel proud that people appreciate me for my work’.

As well this type of service was described by

‘Help to mankind is help to God’.

The community members’ perceptions of the lymphedema management program focused more on the community awareness campaigns which occurred during the program as well as the distribution of the patient kits. A 50 year old housewife explained:

‘We know about the kit which was distributed to the patients like soap, towel, ointment etc.’

A 63 year old farmer in Manibandha described a theatrical awareness campaign:

‘Through Palla (Folk Dance) I learn how to keep the environment clean and use of mosquito net to prevent lymphedema.’

#### Changes in perception of lymphedema during lymphedema management program

Since participating in the lymphedema management program, patients generally emphasized that their communities were accepting of them and did not obstruct their participation in community events. They also noted increased support from the program and appreciation from their families and community members after they observed that the lymphedema management program was helping them:

‘The family and community members also appreciate the work of [the program]’ (74 year old male farmer, unilateral, stage 2).

A 41 year old male laborer (unilateral, stage 2) explained why he thought community members were open to the program:

‘There are no obstacles I faced from my family members or community members. [The program] is helping us so that my next generation will be free from these diseases. ‘

Family members also commented on an improvement of attitudes towards patients who were previously stigmatized. A 45 year old businessman mentioned that

‘Previously people disliked lymphedema patients but now there is no discrimination, people are aware through television, radio about the disease.’

Even so, several males felt that their family members’ lymphedema could still be used as a target during quarrels, but few examples of discrimination were listed. A 70 year old female family member claimed

‘During fever with vomiting people feel very unclean about [accepting Lymphedema patients].

Community members in general insisted that discrimination against lymphedema patients did not exist and all the participants in one male focus group concurred:

‘There is no restriction for them to participate in any village function, they worship with us in the same temple, we bathe in a common pond’.

#### Gender and marriage issues

While most of the focus groups denied significant differences between how patients of different genders experience lymphedema, the issue of marriageability was an area where differences in gender were noted. For example, a 33 year old female family member claimed:

‘Marriage is difficult for a girl if she is a patient. But in case of men sometime it’s possible.’

The concern regarding marriageability among female patients involved not only the possibility of finding a groom, but also the ability to marry someone of suitable social status. A 45 year old male farmer (unilateral, stage 2) noted

‘For a young girl with LF it is very difficult to get married.’

In addition, a 60 year old retired male teacher (community member) stated,

‘It’s difficult to find a groom for a girl [patient] in the same status.’

Issues remained for lymphedema patients who were already married, as demonstrated by two female patients who described having to leave their husbands after their in-laws had found out about their illness. Four other women who discussed their own marriages claimed that their in-laws and/or husbands had been supportive. One 45 year old housewife (bilateral, stage 2) had a more mixed experience, explaining:

‘My relationship with my husband is good but after the disease the relationship with my in-laws has not been good.’

The roles of mothers with lymphedema were not directly explored, but one male family member stated that his children’s studies were affected by his wife’s lymphedema and another male stated:

‘My mother is a patient so we face a lot of problems. If mother is healthy then the entire family is healthy.’

## Discussion

### Knowledge and Local Understanding

These focus groups demonstrated that a basic understanding of LF infection and disease can co-exist with alternative local explanations. Our study adds to previous work from India that demonstrated similar local understandings of causes of LF and lymphedema, [29] specifically diet, hard labor in the field, and heredity. As there are gaps in our understanding of the pathogenesis of filarial lymphedema, it is recognized that immunologic and genetic factors have been implicated in addition to environmental and demographic characteristics. [30] Nevertheless, in other research from India, the study population had not been exposed to education on LF and therefore only 9–20% of respondents identified mosquitoes as the vectors of infection, whereas in this study, we found that in all focus groups there was mention of the mosquito vector. [29] This acceptance of multiple explanations of disease, or cognitive dissonance, is not unique. A study of patients in a rural hospital in South Africa revealed that although most patients held traditional beliefs about illness and treatment, they were accepting of the hospital’s ‘biomedical healthcare’ and believed the two systems could co-exist. Nurses’ acceptance of these beliefs enabled patients to feel comfortable navigating the two systems and reaching compromises. [31] Similar observance of traditional beliefs while continuing to access biomedical care has been observed in antenatal care patients in Zambia and Swaziland. [32]

### Impact of Lymphedema and Acute Episodes on Patients and Family

It is noteworthy that the economic impacts of living with lymphedema were a major theme among patients and family members, emphasizing lost productivity and financial losses associated with lymphedema and acute episodes and costs for treatment. This has also been well documented in other research. [1,25,33] Research at the global level has demonstrated the benefits of the first eight years of the global program to eliminate lymphatic filariasis on increased work productivity amounting to $255 lifetime benefit per individual affected by lymphedema or hydrocele, even when costs associated with social stigma, family members missing work or school to care for patients, reduced quality of life, and lower productivity rates (per day at work) are excluded. [34]

### Experiences and Impact of the Morbidity Management Program

This qualitative study demonstrates how the psychosocial effects and economic impacts experienced by individuals with filarial lymphedema can be ameliorated with the implementation of a community-based lymphedema management program. Similar to previous work, this study shows that lymphedema patients experience significant stigma within their community. [35] The experiences of stigma discussed in these focus groups echoed themes identified by other researchers in Ghana and the Dominican Republic, including feelings of social isolation, marriage issues, teasing and fear of contamination. [12] However given that these focus group discussions occurred years after the start of a community-based lymphedema management program, these topics were interwoven with more positive comments reflecting social acceptance and inclusion coming from the lymphedema patients, as well as family and community members. Previous evaluations of this program have demonstrated clinical improvements in lymphedema and acute episodes, [36] decreases in perceived disability associated with lymphedema, improved productivity, [25] and improved compliance with mass drug administration (MDA). [37] Likely, due to these positive impacts, the community-based lymphedema management program has earned the trust of the community in accepting educational messages on prevention and treatment for LF. A similar effect occurred among lymphedema patients and their communities in a podoconiosis program in Ethiopia, where marked improvements in patient outcomes helped gain acceptance of program messaging. [38]

It was noteworthy that community members seemed eager to emphasize their understanding that the development of lymphedema was not the fault of the patient, but rather considered a preventable and manageable condition. The open and frank manner in which patients, families and, perhaps most importantly, community members, discussed lymphedema in these sessions points to an eroding of this stigma in the community.

### Gender and Marriage Issues

Our focus groups focused on lymphedema, which can affect both men and women, as opposed to hydrocele, which is male-specific. Similar to data from other studies, [12,14] participants in these focus groups emphasized that lymphedema was more difficult for women, given its potential negative impact on marriageability and the implications for family life. This is similar to other research from Odisha on the effects of hydrocele on marriage [13] and speaks to the need for gender-specific support groups to assist lymphedema and hydrocele patients in addressing more intimate, personal issues. These support groups have been established in several countries (Haiti, [39] Brazil, [27] India, [40] Dominican Republic, Ghana [12]) and have had positive impacts for lymphedema and hydrocele patients.

### Limitations

There were several limitations to our study. Although the focus group participants were instructed that their responses were anonymous and in no way would affect their involvement with the program in the future, the focus group facilitators were known to be associated with the non-governmental organization involved in the program. This may have created a social desirability bias where participants may have chosen to give more socially desirable responses to the facilitators. That said, participants did speak freely about their use of alternative medicine or home remedies for lymphedema, although these are not part of the lymphedema management program. There is the possibility for selection bias in this study as well given that those individuals who consented to participate in the discussion groups may be been more knowledgeable about the program or have experienced greater benefit. With translation from the local language, Oriya, to English, we recognize that some nuances associated with participants’ responses may have been lost in translation precluding any detailed analysis of the phrasing or word choice used.

In conclusion, we found that focus group discussions with patients, family, community, and program volunteers expounded upon the more quantitative data gathered on the clinical, quality of life, and productivity benefit of a community-based lymphedema management program. Qualitative research can provide a more nuanced understanding of program benefits and continued patient challenges. Local understandings of disease can co-exist with more biomedical explanations and may not preclude individuals from participating in biomedical interventions. There is a continued need for gender-specific psychosocial support groups to address issues particular to men and women as well as a continued need for improved economic opportunities for LF-affected patients. There is an urgent need to scale-up LF morbidity management and disability prevention programs to reduce the suffering of people affected by LF, which is a global priority.
